# Clonal Hematopoiesis Risk Score and All-Cause and Cardiovascular Mortality in Older Adults

**DOI:** 10.1001/jamanetworkopen.2023.51927

**Published:** 2024-01-17

**Authors:** Seyedmohammad Saadatagah, Md Mesbah Uddin, Lachelle D. Weeks, Abhishek Niroula, Meng Ru, Koichi Takahashi, Lukasz Gondek, Bing Yu, Alexander G. Bick, Benjamin L. Ebert, Elizabeth A. Platz, Pradeep Natarajan, Christie M. Ballantyne

**Affiliations:** 1 Department of Medicine, Baylor College of Medicine, Houston, Texas; 2 Center for Translational Research on Inflammatory Diseases, Baylor College of Medicine, Houston, Texas; 3 Cardiovascular Research Center, Massachusetts General Hospital, Boston, Massachusetts; 4 Broad Institute of MIT and Harvard, Cambridge, Massachusetts; 5 Department of Medicine, Harvard Medical School, Boston, Massachusetts; 6 Department of Medical Oncology, Dana-Farber Cancer Institute, Boston, Massachusetts; 7 Center for Prevention of Progression, Dana-Farber Cancer Institute, Boston, Massachusetts; 8 Department of Laboratory Medicine, Lund University, Sweden; 9 Department of Epidemiology, Johns Hopkins Bloomberg School of Public Health, Baltimore, Maryland; 10 Department of Leukemia, The University of Texas MD Anderson Cancer Center, Houston; 11 Department of Oncology, Johns Hopkins University, and the Sidney Kimmel Comprehensive Cancer Center at Johns Hopkins, Baltimore, Maryland; 12 Department of Epidemiology, Human Genetics and Environmental Sciences, School of Public Health, The University of Texas Health Science Center at Houston, Houston; 13 Division of Genetic Medicine, Department of Medicine, Vanderbilt University, Nashville, Tennessee; 14 Howard Hughes Medical Institute, Boston, Massachusetts

## Abstract

**Importance:**

Clonal hematopoiesis (CH) with acquired pathogenic variants in myeloid leukemia driver genes is common in older adults but of unknown prognostic value.

**Objective:**

To investigate the prevalence of CH and the utility of the CH risk score (CHRS) in estimating all-cause and disease-specific mortality in older adults with CH.

**Design, Setting, and Participants:**

This population-based prospective cohort study involved community-dwelling older adults (aged 67-90 years) without hematologic malignant neoplasms (HMs) who were participants in the Atherosclerosis Risk in Communities Visit 5 at 4 US centers: Forsyth County, North Carolina; Jackson, Mississippi; Minneapolis, Minnesota; and Washington County, Maryland. Samples were collected from 2011 to 2013, sequencing was performed in 2022, and data analysis was completed in 2023.

**Exposure:**

The exposure was a diagnosis of CH. CHRS scores (calculated using 8 demographic, complete blood cell count, and molecular factors) were used to categorize individuals with CH into low-risk (CHRS ≤9.5), intermediate-risk (CHRS >9.5 to <12.5), and high-risk (CHRS ≥12.5) groups.

**Main Outcomes and Measures:**

The primary outcome was all-cause mortality, and secondary outcomes were HM mortality, cardiovascular disease mortality, and death from other causes.

**Results:**

Among 3871 participants without a history of HM (mean [SD] age, 75.7 [5.2] years; 2264 [58.5%] female individuals; 895 [23.1%] Black individuals; 2976 White individuals [76.9%]), 938 (24.2%) had CH. According to the CHRS, 562 (59.9%) were low risk, 318 (33.9%) were intermediate risk, and 58 (6.2%) were high risk. During a median (IQR) follow-up of 7.13 (5.63-7.78) years, 570 participants without CH (19.4%) and 254 participants with CH (27.1%) died. Mortality by CHRS risk group was 128 deaths (22.8%) for low risk, 93 (29.2%) for intermediate risk, and 33 (56.9%) for high risk. By use of multivariable competing risk regression, subdistribution hazard ratios (sHRs) for all-cause mortality were 1.08 (95% CI, 0.89-1.31; *P* = .42) for low-risk CH, 1.12 (95% CI, 0.89-1.41; *P* = .31) for intermediate-risk CH, and 2.52 (95% CI, 1.72-3.70; *P* < .001) for high-risk CH compared with no CH. Among individuals in the high-risk CH group, the sHR of death from HM (6 deaths [10.3%]) was 25.58 (95% CI, 7.55-86.71; *P* < .001) and that of cardiovascular death (12 deaths [20.7%]) was 2.91 (95% CI, 1.55-5.47; *P* < .001).

**Conclusions and Relevance:**

In this cohort study, the CHRS was associated with all-cause, HM-related, and cardiovascular disease mortality in older adults with CH and may be useful in shared decision-making to guide clinical management and identify appropriate candidates for clinical trials.

## Key Points

**Question:**

Is the clonal hematopoiesis (CH) risk score associated with all-cause and disease-specific mortality in older adults with CH?

**Findings:**

In this cohort study that included 3871 individuals without hematologic malignant neoplasms at baseline, 24.2% had CH. Individuals with low-risk CH (59.9% of those with CH) had survival similar to those without CH, and participants with high-risk CH (6.2% of those with CH) had increased all-cause, cardiovascular, and hematologic malignant neoplasm–related mortality.

**Meaning:**

These findings suggest that the CH risk score is associated with overall and disease-specific mortality in older adults with CH and could be used to identify individuals with CH who need more intensive surveillance.

## Teaser

This cohort study investigates prevalence of clonal hematopoiesis and the utility of the clonal hematopoiesis risk score in estimating all-cause and disease-specific mortality among older adults with clonal hematopoiesis.

## Introduction

Clonal hematopoiesis (CH) becomes increasingly common with age.^1^ A subset of CH caused by acquired leukemogenic variants is referred to as CH of indeterminate potential (CHIP), or clonal cytopenia of undetermined significance (CCUS), according to the absence or presence of cytopenia.^2^ CHIP/CCUS was initially described as a risk factor for hematologic (particularly myeloid) malignant neoplasms (HMs). In addition, multiple studies have demonstrated increased mortality and elevated risk of cardiovascular disease, largely among middle-aged adults.^3^ However, the prognostic value of CHIP/CCUS in the older population is understudied.

Recently, the CH risk score (CHRS)—a prognostic tool that uses demographic variables, complete blood cell count parameters, and molecular features—was developed to estimate the risk of myeloid malignant neoplasms in patients with CHIP/CCUS.^4^ In this cohort study, we examine the prognostic value of CHIP/CCUS in a cohort of older adults (aged ≥65 years) and investigate the utility of CHRS in estimating overall and disease-specific mortality.

## Methods

The Atherosclerosis Risk in Communities (ARIC) study and definition of covariates have been previously described (eAppendix in Supplement 1).^5,6^ This study was in accordance with the Strengthening the Reporting of Observational Studies in Epidemiology (STROBE) reporting guidelines for cohort studies. Institutional review boards of all participating centers (Forsyth County, North Carolina; Jackson, Mississippi; Minneapolis, Minnesota; and Washington County, Maryland) approved the study protocol, and all participants provided written informed consent.

For this report, CHIP/CCUS, defined as the presence of somatic variants in myeloid malignant neoplasm driver genes at variant allele frequency greater than or equal to 2%, was identified from analysis of exome sequencing of peripheral blood DNA using the Genome Analysis ToolKit^7^ MuTect2 somatic variant caller, as previously described (eAppendix in Supplement 1).^8^ CHRSs were calculated using 8 factors (eAppendix in Supplement 1) and categorized individuals with CHIP/CCUS into low-risk (CHRS ≤9.5), intermediate-risk (CHRS >9.5 to <12.5), and high-risk (CHRS ≥12.5) groups. Everyone in our cohort received 1.5 points for age. Details and validation of the CHRS were previously described.^4^ Baseline characteristics were tabulated and compared between those with and without CHIP and among CHRS subgroups.

Death was the primary outcome, and cause of death was ascertained using diagnostic codes from hospital discharge records and death certificates (eTable 1 in Supplement 1). Follow-up was from ARIC Visit 5 (V5) (ie, blood draw) until death or administrative censoring (December 31, 2017, for participants from the Jackson, Mississippi, center; December 31, 2019, for other centers).

### Statistical Analysis

We used Fine-Gray competing risk regression to quantify the association between exposures and outcomes.^9^ Covariates were age, sex, race (self-reported), center, hypertension, diabetes, smoking status, coronary artery disease, heart failure, and history of solid cancer. Race was classified as Black or White and was included in this study because of cofounder effects and the association with mortality. Participants with race other than Black or White were excluded from the analysis owing to small numbers. To contextualize relative effects, the risk associated with CHRS risk groups was compared with other clinical conditions. In all comparisons, individuals without CH were considered as reference. Two sensitivity analyses were performed. First, we used inverse probability weighting to evaluate potential selective attrition. In addition, we investigated whether the results derived from the cause of death aligned with adjudicated cardiovascular events (eAppendix in Supplement 1). All statistical analyses were performed using R statistical software version 4.2.2 (R Project for Statistical Computing), and statistical significance was assigned as *P* < .05.

## Results

Of 6538 ARIC-V5 participants (2011-2013), complete blood cell count was available for 6443, and exome sequencing was available for 4233. After excluding 52 participants with HM, 3871 individuals (mean [SD] age, 75.7 [5.2] years; 2264 female individuals [58.5%]; 895 Black individuals [23.1%]; 2976 White individuals [76.9%]) were included for final analysis. We identified 1168 CH variants (eTable 2 in Supplement 1) in 938 individuals (24.2% of the cohort). Of the individuals with CHIP/CCUS, 193 (20.6%) had more than 1 variant (eFigure 1 in Supplement 1), and 108 (11.5%) carried variants in genes defined as high risk by CHRS (eTable 3 in Supplement 1).

Baseline characteristics of those with and without CHIP/CCUS are summarized in Table 1. During the median (IQR) follow-up period of 7.13 (5.63-7.78) years, 254 (27.1%) and 570 (19.4%) deaths occurred in individuals with and without CHIP/CCUS, respectively (subdistribution hazard ratio [sHR], 1.18; 95% CI, 1.01-1.38; *P* = .03). There was no difference in the cumulative incidence of cardiovascular death (86 deaths [9.2%] vs 189 deaths [6.4%]; sHR, 1.17; 95% CI, 0.90-1.53; *P* = .24), but the relative risk of death from HM was higher in individuals with CHIP/CCUS vs those without (17 deaths [1.8%] vs 12 deaths [0.4%]; sHR, 3.97; 95% CI, 1.79-8.80; *P* < .001) (Table 2; eFigure 2 in Supplement 1).

**Table 1.** Baseline Clinical Characteristics of the Study Population by Clonal Hematopoiesis Risk Score Risk Group

Characteristic	Presence of CHIP/CCUS			CHIP/CCUS risk group			
	No (n = 2933)	Yes (n = 938)	*P* value	Low (n = 562)	Intermediate (n = 318)	High (n = 58)	*P* value
Age, mean (SD), y	75.31 (5.06)	76.86 (5.41)	<.001	76.22 (5.25)	77.65 (5.57)	78.76 (5.18)	<.001
Sex							
Male	1172 (40.0)	435 (46.4)	.001	239 (42.5)	165 (51.9)	31 (53.4)	<.001
Female	1761 (60.0)	503 (53.6)		323 (57.5)	153 (48.1)	27 (46.6)	
Race							
Black	674 (23.0)	221 (23.6)	.75	111 (19.8)	96 (30.2)	14 (24.1)	.006
White	2259 (77.0)	717 (76.4)		451 (80.2)	222 (69.8)	44 (75.9)	
Center							
Forsyth County, North Carolina	526 (17.9)	178 (19.0)	.89	111 (19.8)	58 (18.2)	9 (15.5)	.007
Jackson, Mississippi	631 (21.5)	202 (21.5)		100 (17.8)	89 (28.0)	13 (22.4)	
Minneapolis, Minnesota	1061 (36.2)	337 (35.9)		219 (39.0)	99 (31.1)	19 (32.8)	
Washington County, Maryland	715 (24.4)	221 (23.6)		132 (23.5)	72 (22.6)	17 (29.3)	
Body mass index, mean (SD)^a^	28.85 (5.69)	28.53 (5.84)	.14	28.40 (5.36)	28.98 (6.60)	27.30 (5.65)	.07
Diabetes	931 (32.7)	286 (31.7)	.63	169 (31.2)	98 (32.3)	19 (33.9)	.92
Hypertension	2124 (73.4)	717 (77.7)	.01	419 (75.6)	255 (81.5)	43 (76.8)	.02
Smoking							
Current	169 (5.9)	50 (5.5)	.01	33 (6.1)	15 (4.9)	2 (3.5)	.12
Former	1344 (47.1)	473 (52.3)		278 (51.3)	163 (53.3)	32 (56.1)	
Never	1126 (39.4)	305 (33.7)		188 (34.7)	101 (33.0)	16 (28.1)	
Unknown	216 (7.6)	77 (8.5)		43 (7.9)	27 (8.8)	7 (12.3)	
History of coronary artery disease	422 (14.4)	158 (16.8)	.08	85 (15.1)	64 (20.1)	9 (15.5)	.06
History of stroke	109 (3.8)	38 (4.2)	.69	18 (3.3)	19 (6.1)	1 (1.8)	.15
History of heart failure	263 (9.1)	82 (8.9)	.90	36 (6.5)	39 (12.5)	7 (12.3)	.02
History of solid cancer	508 (17.3)	174 (18.6)	.42	100 (17.8)	63 (19.8)	11 (19.0)	.73
Follow-up, median (IQR), y	7.18 (5.67-7.82)	6.91 (5.42-7.68)	<.001	7.10 (5.74-7.76)	6.44 (5.27-7.55)	5.54 (3.58-7.35)	<.001

Abbreviations: CCUS, clonal cytopenia of undetermined significance; CHIP, clonal hematopoiesis of indeterminate potential.

^a^ Body mass index is calculated as weight in kilograms divided by height in meters squared.

**Table 2.** All-Cause and Disease-Specific Mortality by CHRS Risk Group

Cause of death and CHRS risk group	No. of events/No. at risk (%)^a^	sHR (95% CI)^b^	*P* value
All cause			
No CHIP/CCUS	570/2933 (19.4)	1 [Reference]	NA
CHIP/CCUS	254/938 (27.1)	1.18 (1.01-1.38)	.03
Low-risk	128/562 (22.8)	1.08 (0.89-1.31)	.42
Intermediate-risk	93/318 (29.2)	1.12 (0.89-1.41)	.31
High-risk	33/58 (56.9)	2.52 (1.72-3.70)	<.001
Cardiovascular			
No CHIP/CCUS	189/2933 (6.4)	1 [Reference]	NA
CHIP/CCUS	86/938 (9.2)	1.17 (0.90-1.53)	.24
Low-risk	40/562 (7.1)	1.03 (0.72-1.46)	.88
Intermediate-risk	34/318 (10.7)	1.14 (0.78-1.66)	.50
High-risk	12/58 (20.7)	2.91 (1.55-5.47)	<.001
Solid cancer			
No CHIP/CCUS	117/2933 (4.0)	1 [Reference]	NA
CHIP/CCUS	49/938 (5.2)	1.23 (0.88-1.72)	.24
Low-risk	28/562 (5.0)	1.20 (0.80-1.81)	.38
Intermediate-risk	17/318 (5.3)	1.19 (0.70-2.00)	.53
High-risk	4/58 (6.9)	1.76 (0.65-4.77)	.26
Neurologic			
No CHIP/CCUS	80/2933 (2.7)	1 [Reference]	NA
CHIP/CCUS	23/938 (2.5)	0.69 (0.42-1.11)	.12
Low-risk	17/562 (3.0)	0.88 (0.51-1.52)	.64
Intermediate-risk	6/318 (1.9)	0.50 (0.21-1.16)	.11
High-risk	0/58	NA	NA
Respiratory			
No CHIP/CCUS	64/2933 (2.2)	1 [Reference]	NA
CHIP/CCUS	25/938 (2.7)	0.99 (0.62-1.60)	.98
Low-risk	9/562 (1.6)	0.68 (0.34-1.38)	.28
Intermediate-risk	13/318 (4.1)	1.29 (0.69-2.42)	.42
High-risk	3/58 (5.2)	1.74 (0.50-6.05)	.38
Hematologic malignant neoplasms			
No CHIP/CCUS	12/2933 (0.4)	1 [Reference]	NA
CHIP/CCUS	17/938 (1.8)	3.97 (1.79-8.80)	<.001
Low-risk	5/562 (0.9)	2.11 (0.72-6.25)	.18
Intermediate-risk	6/318 (1.9)	4.07 (1.52-10.91)	.005
High-risk	6/58 (10.3)	25.58 (7.55-86.71)	<.001
Infection			
No CHIP/CCUS	13/2933 (0.4)	1 [Reference]	NA
CHIP/CCUS	6/938 (0.6)	1.18 (0.46-2.98)	.73
Low-risk	3/562 (0.5)	1.06 (0.31-3.65)	.92
Intermediate-risk	2/318 (0.6)	1.05 (0.24-4.54)	.95
High-risk	1/58 (1.7)	3.05 (0.35-26.91)	.31
Other			
No CHIP/CCUS	91/2933 (3.1)	1 [Reference]	NA
CHIP/CCUS	47/938 (5.0)	1.43 (1.00-2.04)	.05
Low-risk	26/562 (4.6)	1.39 (0.90-2.15)	.14
Intermediate-risk	15/318 (4.7)	1.25 (0.71-2.20)	.44
High-risk	6/58 (10.3)	2.81 (1.27-6.23)	.01

Abbreviations: CCUS, clonal cytopenia of undetermined significance; CHIP, clonal hematopoiesis of indeterminate potential; CHRS, clonal hematopoiesis risk score; NA, not applicable; sHR, subdistribution hazard ratio.

^a^ There were 19 194.1 person-years in the no CHIP/CCUS group, 3675.0 person-years in the low-risk CHIP/CCUS group, 1942.4 person-years in the intermediate-risk CHIP/CCUS group, and 302.9 person-years in the high-risk group CHIP/CCUS group.

^b^ sHRs were calculated with a Fine-Gray competing risk regression model adjusted for age, sex, race, center, diabetes, smoking, coronary artery disease, heart failure, and solid cancer.

Of those with CHIP/CCUS, the CHRS characterized 562 (59.9%) as low risk, 318 (33.9%) as intermediate risk, and 58 (6.2%) as high risk. There were 128 deaths (22.8%) in the low-risk group (sHR, 1.08; 95% CI, 0.89-1.31; *P* = .42), 93 deaths (29.2%) in the intermediate-risk group (sHR, 1.12; 95% CI, 0.89-1.41; *P* = .31), and 33 deaths (56.9%) deaths in the high-risk group (sHR, 2.52; 95% CI, 1.72-3.70; *P* < .001) (Figure, panel A, and Table 2). The relative risk of death associated with high-risk CHRS was comparable to that of heart failure (sHR, 2.34; 95% CI 1.93-2.85; *P* < .001), the primary clinical risk factor for death (Figure, panel B).

**Figure.**
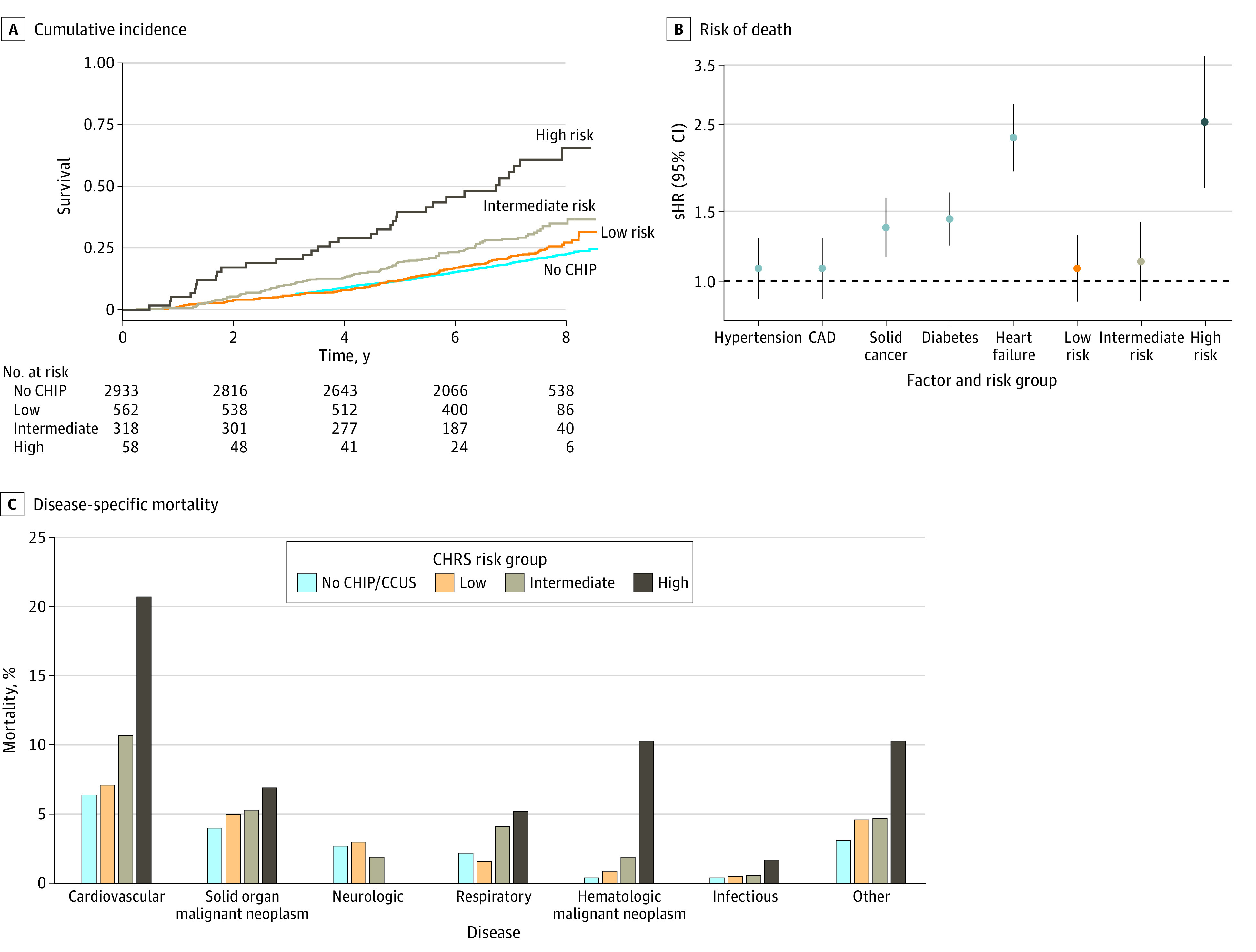
Cumulative Incidence, Associated Risk of Death, and Disease-Specific Mortality by Clonal Hematopoiesis Risk Score (CHRS) Group A, Graph shows cumulative incidence of death according to the CHRS risk group (ie, participants without clonal hematopoiesis [CH] of indeterminate potential [CHIP], with low-risk CH, with intermediate-risk CH, and with high-risk CH). B, Graph shows subdistribution hazard ratios (sHRs) of death associated with clinical risk factors and CHRS risk groups. sHRs were calculated by Fine-Gray competing risk regression, adjusted for age, sex, race, center, hypertension, diabetes, smoking, coronary artery disease, heart failure, and solid cancer. The clinical variable is not included as a covariate when it is the main exposure. C, Graph shows disease-specific mortality by CHRS risk group. CAD indicates coronary artery disease; and CCUS, clonal cytopenia of undetermined significance.

Six people (10.3%) with high-risk CHIP/CCUS died from HM (sHR, 25.58; 95% CI, 7.55-86.71; *P* < .001). However, cardiovascular disease was the most common cause of death in this group, impacting 12 individuals (20.7%) (sHR, 2.91; 95% CI, 1.55-5.47; *P* < .001) (Figure, panel C, and Table 2). Neurologic diseases were the third most common cause of death in individuals without CHIP/CCUS (80 deaths [2.7%]); there was no neurologic disease death in the high-risk group. The findings were consistent in the weighted analysis (eTable 4 in Supplement 1). High-risk CHIP/CCUS was associated with an increased risk of cardiovascular events (eTable 5 in Supplement 1).

## Discussion

CHIP/CCUS is highly prevalent among older adults, and its implications are not well defined. With increased availability of genetic testing for CHIP, extensive evaluation of all older individuals with CHIP/CCUS may overwhelm health care system resources, making identifying individuals with the highest risk of adverse outcomes an attractive endeavor.

In this cohort study of older adults (aged 67-90 years) from ARIC-V5, we showed that the CHRS, a pragmatic prognostic model that uses clinically available data to estimate the risk of myeloid malignant neoplasms in CHIP/CCUS,^4^ was associated with an increased risk of all-cause, HM, and cardiovascular mortality. Notably, everyone in our cohort received 1.5 points for age, resulting in higher proportions of individuals in the intermediate-risk and high-risk CHIP/CCUS groups compared with the original study.^4^ However, most individuals (59.9%) were still identified as low risk by CHRS and had approximately the same survival rate as those without CHIP/CCUS. In contrast, the risk for death greatly increased in the high-risk group.

Our findings support the clinical utility of CHRS in identifying individuals with CHIP/CCUS who need more intensive surveillance for HM. The increased risk for cardiovascular death and cardiovascular events also suggests the need for cardiovascular evaluation and optimization of medical therapies. However, the strategy for risk mitigation in this group is unknown. In addition, clinical trials focusing on novel therapies, designed to enroll participants with high-risk CHIP/CCUS, should carefully adjudicate the incidence of both HM and cardiovascular events.

The inverse association between CHRS categories and neurologic death is consistent with prior reports and merits more investigation.^10,11^ Further studies in larger cohorts of older adults are needed to investigate the generalizability of our findings.

### Limitations

The small number of events, the small number of participants in the high-risk group, left truncation, and residual confounders are the main limitations of the study. The current study solely presents associations, warranting careful consideration when interpreting the findings as a predictive model or drawing causal conclusions.

## Conclusions

In this cohort study of CHIP/CCUS in elderly adults from ARIC-V5, the prevalence of CHIP/CCUS was 24.2%; 59.9% of cases were low risk by CHRS, with about the same survival as those without CHIP/CCUS. In high-risk CHIP/CCUS (6.2% of cases), death from HM had the greatest relative risk, whereas cardiovascular death was the most common cause. The CHRS was associated with all-cause, HM, and cardiovascular disease mortality in older adults with CHIP/CCUS, suggesting that it may be useful in shared decision-making to guide clinical management and identify appropriate candidates for clinical trials.

## Notes

Supplement 1. **eAppendix.** Supplemental Methods
eReferences
**eTable 1.*** ICD* Codes Used to Define Causes of Death **eTable 2.** Clonal Hematopoiesis Driver Gene Frequency in the Study Population **eTable 3.** Distribution of CHRS Components in Participants With CHIP/CCUS and by CHRS Risk Group **eTable 4.** All-Cause and Disease-Specific Mortality by CHRS Risk Group in Inverse Probability of Attrition Weighting Analysis **eTable 5.** Association of CHRS With Incident and Recurrent Cardiovascular Disease **eFigure 1.** Number of Individuals With 1, 2, 3, and ≥4 Variants in the Study Population **eFigure 2.** Cumulative Incidence and Disease-Specific Mortality by CHIP/CCUS Status

Supplement 2.
Data Sharing Statement
