# Supplementary material for: Clonal Hematopoiesis Risk Score and All-Cause and Cardiovascular Mortality in Older Adults
Source: JAMA Netw Open. 2024 Jan 17;7(1):e2351927. doi: 10.1001/jamanetworkopen.2023.51927 (PMC10794939; doi:10.1001/jamanetworkopen.2023.51927)
Supplement: Supplement 1. — eAppendix. Supplemental Methods eReferences eTable 1. ICD Codes Used to Define Causes of Death eTable 2. Clonal Hematopoiesis Driver Gene Frequency in the Study Population eTable 3. Distribution of CHRS Components in Participants With CHIP/CCUS and by CHRS Risk Group eTable 4. All-Cause and Disease-Specific Mortality by CHRS Risk Group in Inverse Probability of Attrition Weighting Analysis eTable 5. Association of CHRS With Incident and Recurrent Cardiovascular Disease eFigure 1. Number of Individuals With 1, 2, 3, and ≥4 Variants in the Study Population eFigure 2. Cumulative Incidence and Disease-Specific Mortality by CHIP/CCUS Status [file jamanetwopen-e2351927-s001.pdf]

Supplemental Online Content

Saadatagah S, Uddin MM, Weeks LD, et al. Clonal hematopoiesis risk score and all-cause and cardiovascular mortality in older adults. *JAMA Netw Open*. 2024;7(1):e2351927. doi:10.1001/jamanetworkopen.2023.51927

eAppendix. Supplemental Methods
eReferences
eTable 1. *ICD* Codes Used to Define Causes of Death
eTable 1. Clonal Hematopoiesis Driver Gene Frequency in the Study Population
eTable 3. Distribution of CHRS Components in Participants With CHIP/CCUS and by CHRS Risk Group
eTable 4. All-Cause and Disease-Specific Mortality by CHRS Risk Group in Inverse Probability of Attrition Weighting Analysis
eTable 5. Association of CHRS With Incident and Recurrent Cardiovascular Disease
eFigure 1. Number of Individuals With 1, 2, 3, and ≥4 Variants in the Study Population
eFigure 2. Cumulative Incidence and Disease-Specific Mortality by CHIP/CCUS Status

This supplemental material has been provided by the authors to give readers additional information about their work.

© 2024 Saadatagah S et al. *JAMA Network Open.*

eAppendix. Supplemental Methods

Covariates
Sex, race, and age were self-reported; a goal of the Atherosclerosis Risk in Communities (ARIC) study is to measure how cardiovascular risk factors, medical care, and outcomes vary by race, sex, place, and time. Height and weight were measured by trained personnel and used to calculate body mass index. Systolic and diastolic blood pressure were measured with an automatic sphygmomanometer at ARIC visit 5 by a certified trained technician using an appropriately sized cuff.[1] An enzymatic assay was used to quantify total cholesterol and high-density lipoprotein cholesterol.[2] Diabetes was defined as fasting serum glucose level ≥126 mg/dL or nonfasting serum glucose level ≥200 mg/dL, self-reported diabetes diagnosed by a physician, or use of hypoglycemic medications. Hematologic malignancies were ascertained using ICD-O-1: 169, 196; ICD-O-2: 42, 77; ICD-O-3: 42, 77; ICD-8: 120.3–120.7; ICD-9:200–208; and ICD-10: 81–86, 88, 90–96. Coronary heart disease and stroke were defined as self-reported myocardial infarction or stroke (ischemic and hemorrhagic) before visit 1 or ARIC-adjudicated myocardial infarction or stroke, silent myocardial infarction identified by ECG changes, or coronary revascularization afterward.[3] Heart failure (HF) was determined by diagnosis code (ICD-9 code 428) or self-reported HF prior to 2005 or by adjudication by an expert panel since 2005.[4]

CHIP ascertainment
Somatic mutations were identified from whole-exome sequencing (WES) using Mutect2 software[5] on the Terra platform (https://portal.firecloud.org/?return=terra#methods/gatk/mutect2-gatk4/21), and CHIP was detected using a publicly available pipeline (https://app.terra.bio/#workspaces/terra-outreach/CHIP-Detection-Mutect2/). To minimize potential artifacts in the Mutect2 calls, a panel-of-normal (PON) was created from 100 random HiSeq WES from the youngest participants, and 1000 Genomes PON was used for the NovaSeq WES. In addition, Genome Aggregation Database (gnomAD) was used to limit germline variants in the somatic mutation call. Mutect2 calls were further filtered, and variants were kept if (i) total depth of coverage ≥20, (ii) number of reads supporting the alternate allele ≥3, (iii) ≥1 read in both forward and reverse direction supporting the alternate allele, (iv) variant allele fraction ≥0.02, (v) gnomAD allele frequency ≤0.001 (not hotspot mutations). Finally, CHIP mutations that passed sequence-based filtering were manually curated. To identify CHIP, pathogenic variants were queried in 69 genes known to drive clonal hematopoiesis and myeloid malignancies.[6-8] The detailed CHIP calling pipeline was previously reported[7,9] (https://app.terra.bio/#workspaces/terra-outreach/CHIP-Detection-Mutect). A special approach was used to identify somatic variants in U2AF1 since an erroneous segmental duplication in the hg38 reference genome resulted in a mapping score of zero for this gene during the sequence alignment from FASTQ to BAM/CRAM. We used a custom script (https://github.com/MMesbahU/U2AF1_pileup) to recover hotspot mutations: S34F, S34Y, R156H, Q157P, and Q157R. A minimum of 5 supporting reads for alternate alleles was required to include a somatic mutation in U2AF1.

CHRS calculation
The clonal hematopoiesis risk score was calculated using 8 factors: a) single *DNMT3A* (present: 0.5 points; absent: 1 point); b) high-risk mutation (absent: 1 point; present: 2.5 points); c) number of mutations (1: 1 point; ≥2: 2 points); d) variant allele frequency (<0.2: 1 point; ≥0.2: 2 points); e) red cell distribution width (<15: 1 point; ≥15: 2.5 points), f) mean corpuscular volume (<100: 1 point; ≥100: 2.5 points); g) cytopenia (absent: 1 point; present: 1.5 points); h) age (<65: 1 point; ≥65: 1.5 points). High-risk mutation was defined as a mutation in splicing factors (*SF3B1*, *SRSF2*, or *ZRSR2*), AML-like genes (*IDH1*, *IDH2*, *RUNX1*, or *FLT3*), *JAK2*, or *TP53*. Cytopenia was defined as hemoglobin <13 g/dL in males or <12 g/dL in females, platelets <150,000 μL^{-1}, or granulocyte absolute count <1800 μL^{-1}.

Addressing survival bias using inverse probability of attrition weighting
We used the inverse probability of attrition weighting (IPAW) method[10] to evaluate and correct the possible effects of selective attrition. We developed a logistic regression model, predicting attrition due to mortality and other loss to follow-up (censoring) from visit 1 to visit 5. The model included age, sex, race * center, hypertension, diabetes, smoking, body mass index, coronary artery disease, HF, and stroke at visit 1. No participants had known solid or hematologic malignancies at the time of enrollment at visit 1. The area under the curve was 0.74. The weight for visit 5 was the inverse of the fitted values (ranging from 1.03 to 5.80) and was added as a covariate to the Fine–Gray competing risk regression model. Other covariates were age, sex, race, center, hypertension, diabetes, smoking status, coronary artery disease, HF, and history of solid cancer.

© 2024 Saadatagah S et al. *JAMA Network Open.*

Studying the association of CHRS with incident/recurrent cardiovascular disease

The association of CHRS with incident/recurrent events was studied using the Fine–Gray competing risk regression model. Cardiovascular disease was defined as the composite endpoint of coronary artery disease, HF, and stroke (defined as above). The event date for participants with no cardiovascular disease at the time of enrollment was the time of the first incident event, and for participants with cardiovascular disease at the time of enrolment was the time of the first recurrent event. The model was adjusted for age, sex, race, center, hypertension, diabetes, and smoking. The event date was considered the endpoint, and participants without outcomes were censored at loss to follow-up or death. The last follow-up date for individuals without events was December 31, 2017, for participants from the Jackson center and December 31, 2019, for other centers.

© 2024 Saadatagah S et al. *JAMA Network Open.*

eReferences

1. Madan N, Lee AK, Matsushita K, et al. Relation of Isolated Systolic Hypertension and Pulse Pressure to High-Sensitivity Cardiac Troponin-T and N-Terminal pro-B-Type Natriuretic Peptide in Older Adults (from the Atherosclerosis Risk in Communities Study). *Am J Cardiol*. Jul 15 2019;124(2):245-252. doi:10.1016/j.amjcard.2019.04.030
2. Sharrett AR, Patsch W, Sorlie PD, Heiss G, Bond MG, Davis CE. Associations of lipoprotein cholesterols, apolipoproteins A-I and B, and triglycerides with carotid atherosclerosis and coronary heart disease: the Atherosclerosis Risk in Communities (ARIC) Study. *Arterioscler Thromb*. Jul 1994;14(7):1098-104.
3. White AD, Folsom AR, Chambless LE, et al. Community surveillance of coronary heart disease in the Atherosclerosis Risk in Communities (ARIC) Study: methods and initial two years' experience. *J Clin Epidemiol*. Feb 1996;49(2):223-33. doi:10.1016/0895-4356(95)00041-0
4. Loehr LR, Rosamond WD, Chang PP, Folsom AR, Chambless LE. Heart failure incidence and survival (from the Atherosclerosis Risk in Communities study). *Am J Cardiol*. Apr 1 2008;101(7):1016-22. doi:10.1016/j.amjcard.2007.11.061
5. Benjamin D, Sato T, Cibulskis K, Getz G, Stewart C, Lichtenstein L. Calling somatic SNVs and indels with Mutect2. *BioRxiv*. 2019:861054.
6. Jaiswal S, Natarajan P, Silver AJ, et al. Clonal hematopoiesis and risk of atherosclerotic cardiovascular disease. *New England Journal of Medicine*. 2017;377(2):111-121.
7. Bick AG, Weinstock JS, Nandakumar SK, et al. Inherited causes of clonal haematopoiesis in 97,691 whole genomes. *Nature*. 2020;586(7831):763-768.
8. Gibson CJ, Lindsley RC, Tchekmedyian V, et al. Clonal hematopoiesis associated with adverse outcomes after autologous stem-cell transplantation for lymphoma. *Journal of Clinical Oncology*. 2017;35(14):1598.
9. Uddin MM, Yu Z, Weinstock JS, et al. Germline genomic and phenomic landscape of clonal hematopoiesis in 323,112 individuals. *medRxiv*. 2022:2022.07. 29.22278015.
10. Weuve J, Tchetgen EJT, Glymour MM, et al. Accounting for bias due to selective attrition: the example of smoking and cognitive decline. *Epidemiology (Cambridge, Mass)*. 2012;23(1):119.

© 2024 Saadatagah S et al. *JAMA Network Open.*

eTable 1. *ICD* Codes Used to Define Causes of Death

Cause of death	ICD9	ICD10
Cardiovascular	393 to 459	I-01 to I-99
Solid cancer	140 to199, 210 to 237,<br>238.[-4, -71 to -76]<br>239	C01 to C80, C-97 to C-99,<br>D-01 to D-44, D-47[-3, -4]<br>D-48, D-49
Neurologic	290 to 389	F-00 to F-99, G-00 to G-99
Respiratory	460 to 519	J-00 to J-99
Hematologic malignancies	200 to 209<br>238.4, 238.71,<br>238.72-238.75, 238.76	C-81 to C-96<br>D45, D46, D473, D474
Infection	001 to 139	A-00 to A-99, B-00 to B-99
Other	All other codes	All other codes

Abbreviation: ICD, international classification of disease.

© 2024 Saadatagah S et al. *JAMA Network Open.*

eTable 2. Clonal Hematopoiesis Driver Gene Frequency in the Study Population

Rank	Gene	Number
1	*DNMT3A*	499
2	*TET2*	230
3	*ASXL1*	84
4	*PPM1D*	39
5	*SF3B1*[a]	36
6	*SRSF2*[a]	28
7	*TP53*[a]	25
8	*ZNF318*	24
9	*ZBTB33*	19
10	*STAG2*	16
11:12	*SRCAP, YLPM1*	13
13:14	*JAK2,*[a] *U2AF1*	12
15	*BRCC3*	11
16	*PDS5B*	10
17:19	*CBL, GNB1, ZRSR2*[a]	8
20:22	*ASXL2, IDH2,*[a] *NF1*	6
23:25	*KDM6A, PHIP, PRPF8*	5
26	*NRAS*	4
27:30	*CREBBP, CUX1, KRAS, PTEN*	3
31:36	*BCOR, BRAF, EP300, EZH2, NXF1, PHF6*	2
37:48	*BCORL1, CTCF, ETV6, IDH1,*[a] *IKZF2, MPL, PTPN11, RAD21, RUNX1,*[a] *SETBP1, SETD2, SUZ12*	1

[a] High-risk genes.

© 2024 Saadatagah S et al. *JAMA Network Open.*

eTable 3. Distribution of CHRS Components in Participants With CHIP/CCUS and by CHRS Risk Group

CHRS Component	CHIP/CCUS (n = 938)	Low-Risk CHIP/CCUS (n = 562)	Intermediate-Risk CHIP/CCUS (n = 318)	High-Risk CHIP/CCUS (n = 58)
Single *DNMT3A*				
Present (0.5 points)	346 (35.9)	292 (52.0)	54 (17.0)	0 (0.0)
Absent (1.0 point)	592 (63.1)	270 (48.0)	264 (83.0)	58 (100.0)
High risk mutation				
Absent (1.0 point)	830 (88.5)	562 (100)	248 (88.0)	20 (34.5)
Present (2.5 points)	108 (11.5)	0 (0.0)	70 (22.0)	38 (65.5)
Number of mutations				
1 (1point)	745 (79.4)	533 (94.8)	203 (63.8)	9 (15.5)
≥ 2 (2 points)	193 (20.6)	29 (5.2)	115 (36.2)	49 (84.5)
VAF				
< 0.2 (1 point)	702 (74.8)	496 (88.3)	199 (62.6)	7 (12.1)
≥ 0.2 (2 points)	236 (25.2)	66 (11.7)	119 (37.4)	51 (87.9)
Age				
< 65 (1 point)	0 (0.0)	0 (0.0)	0 (0.0)	0 (0.0)
≥ 65 (1.5 points)	938 (100.0)	562 (100.0)	318 (100.0)	58 (100.0)
RDW				
< 15 (1 point)	685 (73.0)	532 (94.7)	139 (43.7)	14 (24.1)
≥ 15 (2.5 points)	253 (27.0)	30 (5.3)	179 (56.3)	44 (75.9)
MCV				
< 100 (1 point)	899 (95.8)	559 (99.5)	293 (92.1)	47 (81.0)
≥ 100 (2.5 points)	39 (4.2)	3 (0.5)	25 (7.9)	11 (19.0)
Cytopenia				
Absent (1.0 point)	612 (65.2)	433 (77.0)	167 (52.5)	12 (20.7)
Present (1.5 points)	326 (34.8)	129 (23.0)	151 (47.5)	46 (79.3)

Data are presented as n (%).
Abbreviations: CCUS, clonal cytopenia of undetermined significance; CHIP, clonal hematopoiesis of indeterminate potential; CHRS, clonal hematopoiesis risk score; MCV, mean corpuscular volume; RDW, red cell distribution width; VAF, variant allele frequency.

© 2024 Saadatagah S et al. *JAMA Network Open.*

eTable 4. All-Cause and Disease-Specific Mortality by CHRS Risk Group in Inverse Probability of Attrition Weighting Analysis

CHRS Risk Group	Death	#at risk[a] | #events (%)	sHR (95% CI)[b]	*P*
No CHIP/CCUS	All cause	2933 | 570 (19.4)	Ref	-
CHIP/CCUS	All cause	938 | 254 (27.1)	1.18 (1.01 - 1.38)	0.03
Low-Risk	All cause	562 | 128 (22.8)	1.08 (0.89 - 1.31)	0.42
Intermediate-Risk	All cause	318 | 93 (29.2)	1.13 (0.90 - 1.42)	0.29
High-Risk	All cause	58 | 33 (56.9)	2.52 (1.71 - 3.69)	<.001
No CHIP/CCUS	Cardiovascular	2933 | 189 (6.4)	Ref	-
CHIP/CCUS	Cardiovascular	938 | 86 (9.2)	1.17 (0.90 - 1.53)	0.24
Low-Risk	Cardiovascular	562 | 40 (7.1)	1.03 (0.73 - 1.47)	0.86
Intermediate-Risk	Cardiovascular	318 | 34 (10.7)	1.14 (0.79 - 1.67)	0.48
High-Risk	Cardiovascular	58 | 12 (20.7)	2.91 (1.55 - 5.47)	0.001
No CHIP/CCUS	Solid cancer	2933 | 117 (4.0)	Ref	-
CHIP/CCUS	Solid cancer	938 | 49 (5.2)	1.23 (0.88 - 1.72)	0.24
Low-Risk	Solid cancer	562 | 28 (5.0)	1.20 (0.79 - 1.80)	0.39
Intermediate-Risk	Solid cancer	318 | 17 (5.3)	1.18 (0.70 - 1.99)	0.53
High-Risk	Solid cancer	58 | 4 (6.9)	1.81 (0.67 - 4.85)	0.24
No CHIP/CCUS	Neurologic	2933 | 80 (2.7)	Ref	-
CHIP/CCUS	Neurologic	938 | 23 (2.5)	0.69 (0.42 - 1.11)	0.12
Low-Risk	Neurologic	562 | 17 (3.0)	0.87 (0.50 - 1.50)	0.62
Intermediate-Risk	Neurologic	318 | 6 (1.9)	0.50 (0.21 - 1.16)	0.10
High-Risk	Neurologic	58 | 0 (0.0)	NA	NA
No CHIP/CCUS	Respiratory	2933 | 64 (2.2)	Ref	-
CHIP/CCUS	Respiratory	938 | 25 (2.7)	0.99 (0.62 - 1.60)	0.98
Low-Risk	Respiratory	562 | 9 (1.6)	0.68 (0.33 - 1.37)	0.28
Intermediate-Risk	Respiratory	318 | 13 (4.1)	1.29 (0.69 - 2.40)	0.42
High-Risk	Respiratory	58 | 3 (5.2)	1.71 (0.49 - 5.97)	0.40
No CHIP/CCUS	HM	2933 | 12 (0.4)	Ref	-
CHIP/CCUS	HM	938 | 17 (1.8)	3.97 (1.79 - 8.8)	0.001
Low-Risk	HM	562 | 5 (0.9)	2.11 (0.71 - 6.24)	0.180
Intermediate-Risk	HM	318 | 6 (1.9)	4.07 (1.52 - 10.92)	0.005
High-Risk	HM	58 | 6 (10.3)	25.78 (7.52 - 88.34)	<.001
No CHIP/CCUS	Infection	2933 | 13 (0.4)	Ref	-
CHIP/CCUS	Infection	938 | 6 (0.6)	1.18 (0.46 - 2.98)	0.73
Low-Risk	Infection	562 | 3 (0.5)	1.06 (0.31 - 3.66)	0.92
Intermediate-Risk	Infection	318 | 2 (0.6)	1.05 (0.24 - 4.59)	0.95
High-Risk	Infection	58 | 1 (1.7)	3.01 (0.34 - 26.96)	0.32
No CHIP/CCUS	Other	2933 | 91 (3.1)	Ref	-
CHIP/CCUS	Other	938 | 47 (5.0)	1.43 (1.00 - 2.04)	0.05
Low-Risk	Other	562 | 26 (4.6)	1.39 (0.90 - 2.15)	0.14
Intermediate-Risk	Other	318 | 15 (4.7)	1.26 (0.72 - 2.21)	0.43
High-Risk	Other	58 | 6 (10.3)	2.80 (1.26 - 6.22)	0.01

[a] The person-years for No CHIP/CCUS, Low-Risk, Intermediate-Risk, and High-Risk groups are 19194.1, 3675.0, 1942.4, and 302.9, respectively.
[b] Fine–Gray competing risk regression model, adjusted for age, sex, race, center, diabetes, smoking, coronary artery disease, heart failure, solid cancer, and inverse probability of attrition weighting from visit 1 to visit 5.
Abbreviation, CCUS, clonal cytopenia of undetermined significance; CHIP, clonal hematopoiesis of indeterminate potential; CHRS, clonal hematopoiesis risk score; CI, confidence interval; HM, hematologic malignancy; sHR, subdistribution hazard ratio.

© 2024 Saadatagah S et al. *JAMA Network Open.*

eTable 5. Association of CHRS With Incident and Recurrent Cardiovascular Disease

CHRS Risk Group	Event	#at risk[a] | #events (%)	sHR (95% CI)[b]	*P*
No CHIP/CCUS	Inc/Rec CVD	2933 | 595 (20.3)	Ref	-
CHIP/CCUS	Inc/Rec CVD	938 | 230 (24.5)	1.07 (0.91 – 1.24)	0.420
Low-Risk	Inc/Rec CVD	562 | 116 (20.6)	0.93 (0.77 – 1.14)	0.500
Intermediate-Risk	Inc/Rec CVD	318 | 93 (29.2)	1.21 (0.97 – 1.52)	0.095
High-Risk	Inc/Rec CVD	58 | 21 (36.2)	1.49 (0.99 – 2.26)	0.006

[a] The person-years for No CHIP/CCUS, Low-Risk, Intermediate-Risk, and High-Risk groups are 19194.1, 3675.0, 1942.4, and 302.9, respectively.
[b] Fine–Gray competing risk regression model is adjusted for age, sex, race, center, hypertension, diabetes, and smoking. Cardiovascular disease is defined as coronary artery disease, heart failure, and stroke.
Abbreviation, CCUS, clonal cytopenia of undetermined significance; CHIP, clonal hematopoiesis of indeterminate potential; CHRS, clonal hematopoiesis risk score; CI, confidence interval; CVD, cardiovascular disease; Inc/Rec, Incident/Recurrent; sHR, subdistribution hazard ratio.

© 2024 Saadatagah S et al. *JAMA Network Open.*

eFigure 1. Number of Individuals With 1, 2, 3, and ≥4 Variants in the Study Population

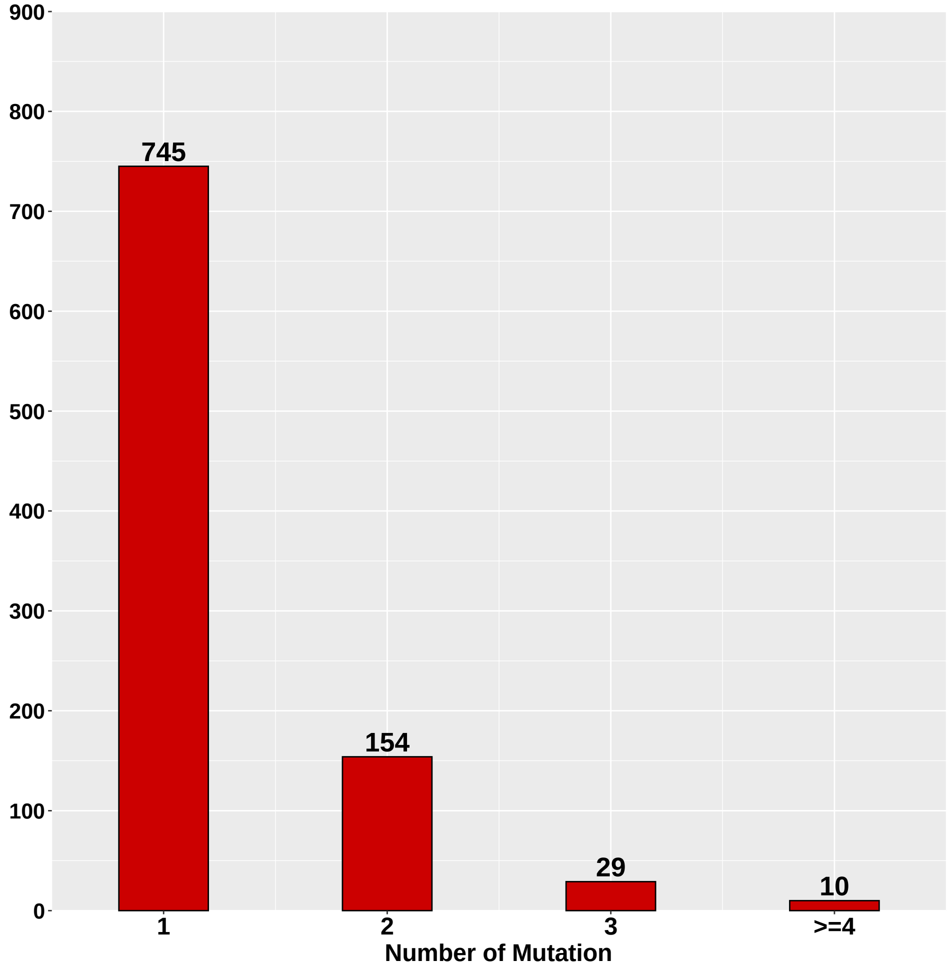

© 2024 Saadatagah S et al. *JAMA Network Open.*

eFigure 2. Cumulative Incidence and Disease-Specific Mortality by CHIP/CCUS Status

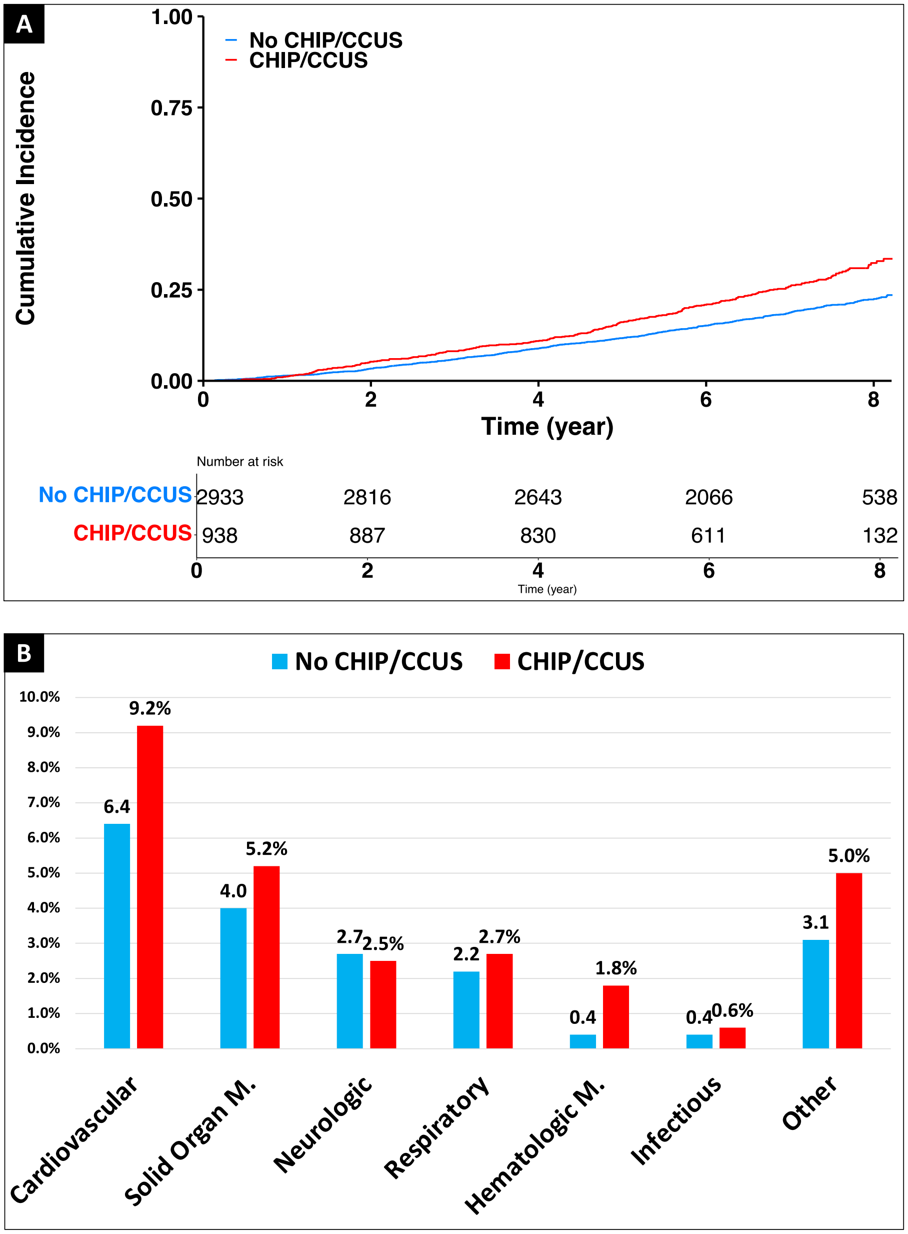

A) Cumulative incidence of death based on CHIP/CCUS status **B**) disease-specific mortality by CHIP/CCUS status.
Abbreviations: CHIP, clonal hematopoiesis. of indeterminate potential; CCUS, clonal cytopenia of undetermined significance.

© 2024 Saadatagah S et al. *JAMA Network Open.*
