# Supplementary material for: Clonal Hematopoiesis Risk Score and All-Cause and Cardiovascular Mortality in Older Adults
Source: JAMA Netw Open. 2024 Jan 17;7(1):e2351927. doi: 10.1001/jamanetworkopen.2023.51927 (PMC10794939; doi:10.1001/jamanetworkopen.2023.51927)
Supplement: Supplement 2. — Data Sharing Statement [file jamanetwopen-e2351927-s002.pdf]

Data Sharing Statement

Saadatagah. Clonal Hematopoiesis Risk Score and All-Cause and Cardiovascular Mortality in Older Adults. *JAMA Netw Open*. Published January 17, 2024.
doi:10.1001/jamanetworkopen.2023.51927

Data
Data available: Yes
Data types: Deidentified participant data
How to access data: https://biolincc.nhlbi.nih.gov/studies/aric/
When available: With publication

Supporting Documents
Document types: None

Additional Information
Who can access the data: researchers must be registered on this site
Types of analyses: https://biolincc.nhlbi.nih.gov/studies/aric/
Mechanisms of data availability: https://biolincc.nhlbi.nih.gov/studies/aric/
